# Poor Provision of Sanitary Facilities in Markets of Lusaka District Zambia

**DOI:** 10.5334/aogh.3400

**Published:** 2021-11-26

**Authors:** Chisala D. Meki, Magne Bråtveit, Charles Michelo, Bente E. Moen

**Affiliations:** 1 University of Zambia, School of Public Health, Dept. of Environmental Health, Lusaka, Zambia; 2 University of Bergen, Dept. of Global Public Health & Primary Care, Bergen, Norway; 3 University of Zambia, Dept. of Epidemiology & Biostatistics, School of Public Health, Lusaka, Zambia; 4 Harvest Research Institutes, Harvest University, Lusaka, Zambia; 5 University of Bergen, Dept. of Global Public Health & Primary Care, Centre for International Health, Bergen, Norway

## Abstract

**Background:**

Although provision of sanitary facilities in workplaces is an important issue, very few studies have been undertaken in this regard.

**Objective:**

This study assessed the provision of sanitary facilities for market traders and their perceptions of the provided facilities in Lusaka district Zambia.

**Methods:**

A cross-sectional study of workplace observations in 12 randomly selected markets and interviews with 386 traders, conducted in Lusaka district.

**Findings:**

The study revealed that eleven of the twelve markets provided toilets, hand-washing and drying facilities, water, urinals, soap, and toilet paper. However, most of the markets did not comply with the Zambian laws in terms of the adequacy and privacy of facilities. One market did not have any of the listed facilities. Most traders perceived facilities to be unsatisfactory and used them only because of the lack of alternatives. Poor provision of sanitary facilities was observed at markets thus predisposing its workforce and trading population to multiple public health hazards.

**Conclusions:**

The findings of this study call for urgent investments in sanitary structures and surveillance systems to guarantee the safety of the population and to promote the health of market traders as well as the community at large.

## Introduction

About 4.5 and 2.1 billion people lack safely managed sanitation and drinking water services, respectively, around the world. Developing countries are most affected by water and sanitation deficiencies [1]. Water, sanitation and hygiene are important for good health, economic and social development [2]. According to the World Health Organization (WHO) “Lack of sanitation contributes to about 10% of global disease burden causing mainly diarrheal diseases” [3]. Sanitation is important not only to prevent endemic diarrhea but also to prevent intestinal helminthiases, giardiasis, schistosomiasis, trachoma and other globally important issues, including malnutrition. Sanitation related diseases weigh heavily on households and health systems. For example, it has been estimated that the health costs alone amount to some US$340 million for households lacking water supply and sanitation and US$7 billion on national health systems [4].

It is a well-known fact that sanitary facilities are an important aspect of everyday life. For example, toilets are important for health, privacy and dignity, while washing facilities are required for personal hygiene [5,6]. The provision of sanitary facilities for workers is particularly important [7]. This is because poor sanitary facilities can affect workers in several ways, for instance by contributing to the spread of diseases such as cholera, dysentery, typhoid and urinary tract infection. This can ultimately lead to fatigue, poor health and death. Poor health may also lead to absenteeism at work which could result in loss of revenue for both the worker and the employer. Lack of a good working environment may also demotivate workers and thereby reduce productivity. Literature indicates that people in general, including workers, are not pleased with facilities that are unclean, not well maintained, inadequate, located at far distances and do not offer privacy and security [8,9,10]. Gender differences exist in perceptions of sanitary facilities, with women seeming to be more concerned with poor facilities than men [11,12,13,14,15,16].

In Zambia, about 66% and 44% of the population have access to safe drinking water and sanitation, respectively [17]. According to the Centre for Disease Control and Prevention (CDC), diarrhea is among the 10 major killer diseases in Zambia [18]. Reports indicate that Zambian workers in markets and schools are exposed to poor sanitation and lack of safe drinking water [19,20,21]. In Zambia, there are several laws that have relevance to the provision of sanitary facilities in workplaces, including the Public Health Act, Factory Act, Occupational Health and safety Act, and Markets and Bus Station Act [22,23,24,25]. These laws define the type, conditions and standards of facilities to be provided at workplaces. For example, the Market and Bus Station Act requires all markets to have some form of sanitary facilities for traders provided by the local authority [24]. Markets are part of the informal workplaces in Zambia and are usually run by the local authorities [24,26,27].

Despite the laws that govern provision of sanitary facilities in Zambia, local reports indicate that some workplaces lack proper sanitary facilities for workers, as already described. However, to our knowledge there are no published studies that have quantified this problem. The problem of poor provision of sanitary facilities is particularly problematic in Lusaka district, where water, sanitation and hygiene are challenging problems. The city is characterized by poor solid waste management, poor drainage facilities and poor water supply. Street vending featuring cooked food is also common, along with deplorable sanitation, especially in peri urban areas and markets [28]. Most of the people in peri-urban areas of Lusaka use poorly constructed pit latrines and open defecation remains a problem [29].

The objectives of this study were therefore to assess the provision of sanitary facilities for market traders of Lusaka district and market traders’ perceptions of the provided facilities. Markets were picked as study areas as Zambia has suffered recurrent diarrhoea-related diseases—especially cholera, with major outbreaks since the 1990s—as a possible consequence of contamination in crowded places such as markets [30,31].

## Material and Methods

### Study design and setting

This study employed a descriptive cross-sectional study design comprising workplace observations and interview of market traders.

### Study setting

**Figure 1:**
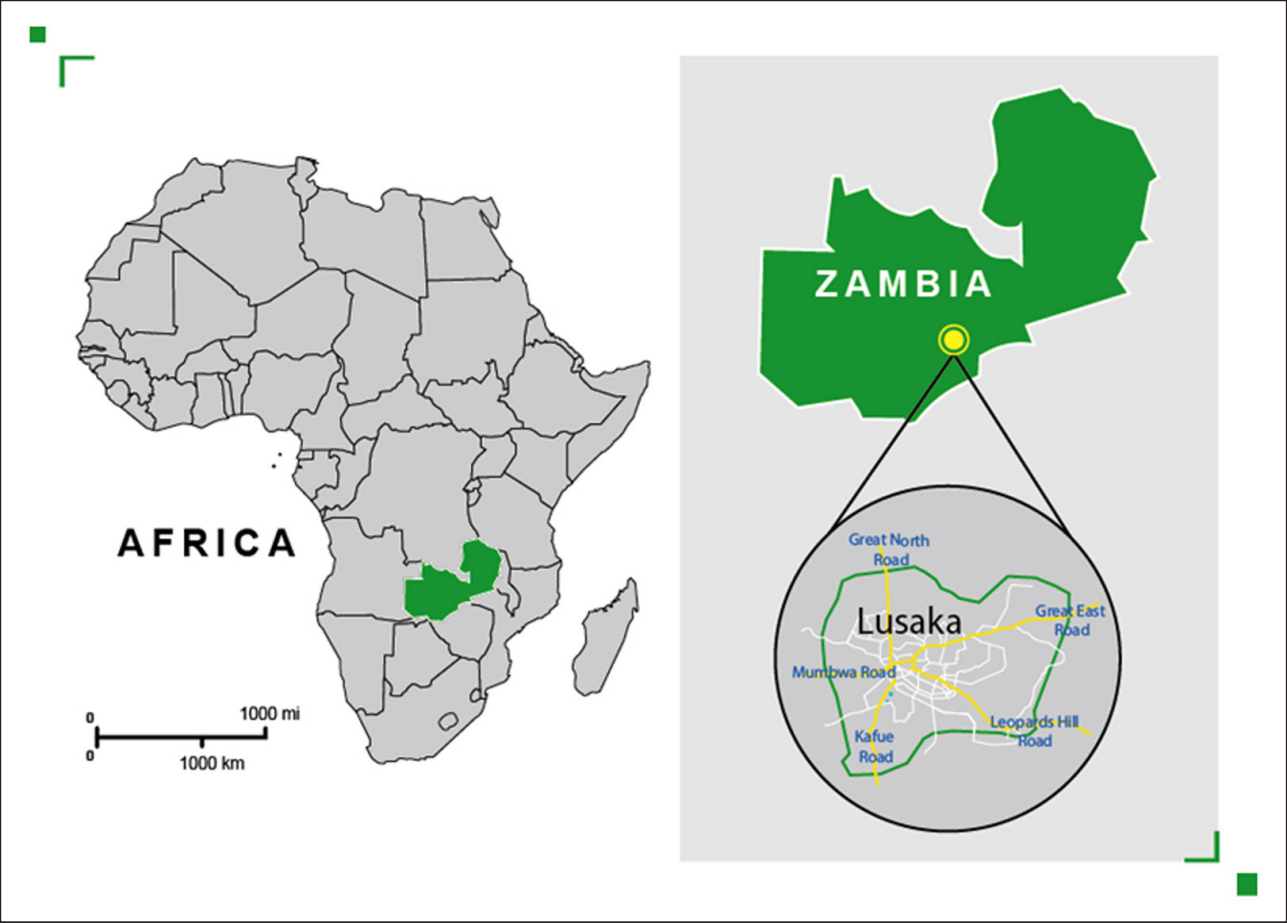
Map of Africa showing Zambia and Lusaka.

**Figure 2:**
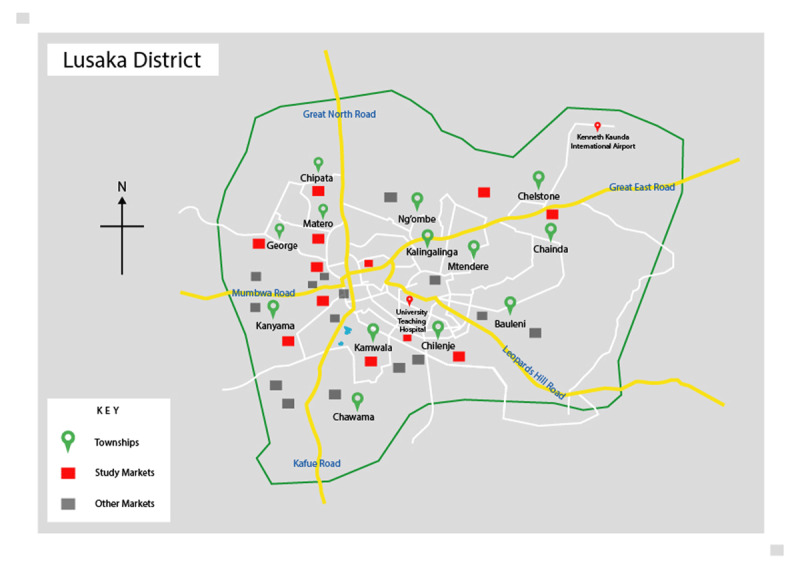
Map of Lusaka District showing the townships and markets.

The study was carried out from September to November 2014 in 12 markets of Lusaka district, the capital and most populated city of Zambia. The population of the city is estimated to be just over 1.7 million people [32]. ***Figures 1*** and ***2*** show the location of the study area.

### Participants

The study included two sets of study units: the markets and traders from the sampled markets of Lusaka district Zambia.

### Eligibility criteria

All the markets run by the city council in Lusaka were eligible to take part in the study. From the markets, all market traders 18 years and above at the time of the study were considered as potential participants.

### Selection of participants

The selection of participants was done in two stages: (1) the market and (2) the market traders.

### Markets

At the time of the study, there were 27 markets in Lusaka run by the local authority—the Lusaka City Council. It is important to note that it was the responsibility of the government through the Lusaka City Council to provide sanitary facilities in the markets. To get the markets to participate in the study the markets were distributed in three categories: central business (eight markets), township (nine markets) and peri-urban areas (10 markets) [20]. A total of 12 markets, four from each of the three market categories, were selected. Four markets from each category were included for convenience to ensure equal representation from the three market categories. To select the actual markets to be included from each category, we used the lottery method for a simple random sampling of the markets. The lottery was conducted as follows: all the names of the markets were written on pieces of paper and put in a holder. The holder was then shaken, and one slip of paper at a time was picked from the holder until 4 (four) markets had been selected from each category.

### Market traders

The number of traders selected from each category of markets was determined using sampling proportionate to size i.e., according to the number of stalls representing the number of traders within each category. The number of traders selected from each of the market categories were 60, 104 and 220 from peri-urban, township and central business, respectively. These market traders were selected using systematic random sampling from market stall lists obtained from each market.

### Variables

The study involved two set of variables those used in the observation and those employed in the interviews of market traders.

### Observed variables

The variables in the observations included availability of facilities, privacy, cleanliness, and requirements for adequacy. The way these variables were measured, and the standards used is presented in ***Table 1***.

**Table 1:** Categorization of observed variables.


VARIABLE	INFORMATION FOR OBSERVATIONS	BASED ON

**Availability**	**Availability** of the following: Toilets, hand washing facilities, washing facilities, hand drying facilities, source of water, urinals, sanitary bins, and tissue	Public Health Act Cap 295 and Literature

**Privacy**	**Private**: All the toilets’ cubicles had lockable doors **Not - private**: Some or all toilets’ cubicles with non-lockable doors or Some or all toilet cubicle without doors	These checkpoints are the researcher’s interpretation of the acts since they do not specify of privacy and cleanliness

**Cleanliness**	**Not clean(poor)**: Presence of water, urine or faecal matter on floor and/or wall, plus offensive smells and flies. **Clean(good)**: No water, urine or faecal matter on floor and/wall, plus absence of offensive smells and flies.	

**Requirements for toilets and wash hand basins PHA**	**Adequate**: Number of toilets and hand washing basins: 1–25 workers should be provided with one (1) latrine (water closet) up to a maximum of the first 100 and then greater than (>) 100 workers one (1) added latrine for every 40 workers. In addition, hand wash basins and urinals must be provided for each water closet provided. **Inadequate**: Number of toilets and hand washing basins: more than 25 workers using 1 single latrine (water closet) up to a maximum of the first 100 and then greater than (>) 100 workers no added latrine for every 40 workers. With no additional hand wash basins and urinals provided for each water closet provided.	The Public Health Act Cap 295


*Note*: Provision of facilities was measured as yes(available) and no (not available). **PHA**: Public Health Act.

### Interview’s variables

The interview variables used in this study were categorized as follows: Toilets and hand washing facilities in terms of numbers (adequate or not adequate), privacy (private or not private), cleanliness (good or poor), siting of facilities (near or far), and use of facilities (always, sometimes or do not use). Other variables included age and sex of traders and type of business.

### Data sources and measurements

The study involved two methods of data collection, including workplace observations and interviews. Workplace observations using checklists were used to obtain information on the provided sanitary facilities in the markets and their conditions. The interviews were used to collect data on the perception of market traders concerning the provided facilities.

### Workplace observations

Non-participant observations of the sanitary facilities provided at each market were done between 10 and 12am by the principal investigator at all markets during the study period, with a focus on selected checkout points. The checkpoints used in the workplace observations were based on a checklist from Nansereko [33], and on the legal requirements for sanitary facilities in Zambia (***Table 1***). Three observations of all the sanitary facilities available in each market were done at two-week intervals between each observation. The researchers decided to have three observations because no standards were found on how many observations would be sufficient for drawing a conclusion. The researchers concluded that three would be enough. The people responsible for running the markets were not informed of the observation days to ensure that they had no opportunity to alter the usual state of the sanitary facilities. Moreover, it was not necessary to alert the market leaders of the observer’s visits and inspection of the facilities because the Occupational Health and Safety Act of 2010 stipulates simply that the employers must “Provide and maintain a working environment for the employees that is, so far as is reasonably practicable, safe and without any risks to their health and safety, and which is adequate as regards facilities and arrangements for their welfare at the workplace” [25].

According to the WHO, universal access to safely managed water, sanitation services and handwashing facilities with soap and water must be guaranteed for all by 2030 [1]. This target is in line with the Sustainable Development Goals (SDGs), and all institutions, including workplaces, must aim to have safely managed drinking water and sanitation services. This means that every workplace should have drinking water from an improved water source located on the premises, available when needed and free of faecal and priority chemical contamination. Safely managed sanitation services must also be provided where excreta are safely disposed of in situ or transported and treated offsite. In addition, basic handwashing facilities must be provided, with soap and water available on the premises.

In this study, water, sanitation and hand washing facilities were classified as either improved or unimproved. Improved water sources consisted of those designed and constructed with the potential to deliver safe water including piped water and boreholes. Unimproved water sources included unprotected dug wells. In terms of sanitation, improved sanitation facilities included flush/pour flush toilets to piped sewer systems, septic tanks and pit latrines with slabs, while unimproved sanitation referred to pit latrines without a slab or platform, bucket latrines (use of shake packs) and open defecation. With respect to handwashing facilities, wash hand basins, buckets with tap(s), and standpipes or tippy taps with water and soap were classified as improved, whereas those without water and soap were classified as unimproved [1].

### Interviews of market traders

The principal investigator collected data on provided sanitary facilities and market traders’ perception of these, with the help of a research assistant trained in the correct way to collect data using semi structured questionnaires. The selected market traders were provided with written information about the study before being interviewed, and traders who agreed to participate in the study signed a consent form. The traders were interviewed using an interview guide, where most of the questions were adopted from a thesis by Nansereko on sanitation in schools [33]. The study aimed to assess the adequacy and utilization of sanitation facilities in secondary schools in Mpigi district.

The systematic interview guide included the following items: basic information of the traders including sex, age and type of business; provided facilities, including adequacy of toilets and hand washing facilities; siting of facilities in relation to the market traders’ stalls, cleanliness of facilities, privacy of facilities and finally, use of facilities.

The full interview schedule and checklist used in this study are available in the full thesis related with this publication [34].

### Bias

The fact that not all markets and traders were included in the study entailed the possibility of selection bias. To control for this bias, all the markets managed by the Lusaka City Council in Lusaka district were included in the study at the initial sampling stage for full representation. The markets were then stratified into categories based on their size and location to make sure that all the groups with different characteristics were represented. Simple random sampling using the lottery method was then used to select the markets to be included in the study from each category because this method ensures that all the markets have the same chance of being included in the study. In the selection of the market traders, we controlled for bias first by sampling by size to ensure that each market was well represented. In addition, market traders to be included in the study were selected using a systematic random sampling using the list of stalls as the sampling frame to ensure that all the traders in each market had the same chance of being included in the study. The trader randomly found at the stall at the time of participant recruitment was included in the study. If more than one person was found at the stall during recruitment of participants, we used simple random sampling by the lottery method to recruit one participant.

### Study size

The sample size for markets was decided on conveniently. The study involved 12 markets. The sample size of market traders (384) was determined using the formula for cross sectional or prevalence studies shown below.


n = \frac{{{Z^2}\;P\left({1 - P} \right)}}{{{d^2}}}

Where:

n = sample size, Z = Z statistic for a level of confidence, P = expected prevalence or proportion (that can be obtained from similar studies, or a pilot study conducted by the researchers) (in proportion of one; if 50%, P = 0.5), and d = precision (corresponding to effect size) (in proportion of one; if 5%, d = 0.05).

In this study, the level of confidence used was 95% with a corresponding Z of 1.96. The P Prevalence (proportion) used in the calculation was the expected prevalence of markets traders without access to sanitation facilities, a P of 50% was used as no pilot study was conducted nor similar studies were identified to obtain the P. The d precision (corresponding to effect size) of 5% was used as the expected prevalence used was within the range of 10 to 90% as recommended in literature [35].

### Data analysis

IBM SPSS version 16 software was used for data entry and analysis. Descriptive statistics, including frequencies and proportions, were obtained. Data were analyzed at market and traders’ levels using observed and interview data, respectively. Missing data were reported independently in the results presentation for each variable. A pilot study that aimed at validating the instruments was done at one of the markets prior to the main study, and data were not included in the analysis. The interview guide was piloted among 10 market traders from a market not included in this study. The point was to check if the questions were understandable, and that the interview did not take too much time. We changed the wording of a few questions in the interview due to this pre-test.

### Ethics and consent

We applied for ethical approval from the Committee for Medical and Health Research Ethics West in Norway (REK West)—Ref. No. 2014/816/REK west—and Excellence in Research Ethics and Science (ERES)—Ref. No. 2014-July-018—in Zambia before data collection. Further, additional consent to conduct the study was obtained from Norway, as the study was carried out in collaboration with the Centre for International Health at the University of Bergen in Norway. We applied to Lusaka City Council for administrative consent to conduct the study and collect information. In addition, market managers and individual market traders gave their consent. Moreover, all consents applied to the plan to carry out a pilot study.

## Results

### Characteristics of study sample

A total of 12 markets were included in the study, four from each of the categories, which included: townships, peri-urban and central business. Stalls within these market categories numbered 1660, 1557 and 3844, respectively (***Tables 2*** and ***3***). A total of 386 market traders participated in the study, 46% men and 54% women. The age of 316 the participants ranged from 18–74 years with the average age being 35 years. In terms of the type of 317 business, 42% were dealing with food and groceries, 33% in beauty, 19% in stationery and hardware, 318 in electronics and the remaining 4% in other business, with 1% missing.

**Table 2:** Provided sanitary facilities in markets of Lusaka district.


MARKET CATEGORY AND NUMBER	FACILITY									

	TOILET	HAND WASHING	WASHING	HAND DRYING	WATER SOURCE	TYPE OF WATER SOURCE	URINAL	SANITARY BIN	SOAP	TISSUE

**Township**										

1	Yes	Yes	Yes	Yes	Yes	Council	Yes	Yes	No	Yes

2	Yes	Yes	Yes	Yes	Yes	Council	Yes	Yes	No	Yes

3	Yes	Yes	Yes	Yes	Yes	Council	Yes	No	No	Yes

4	Yes	Yes	Yes	No	Yes	Council	Yes	No	No	Yes

**Peri-Urban**										

1	Yes	Yes	Yes	No	Yes	Council	Yes	No	No	Yes

2	Yes	Yes	Yes	No	Yes	Council	Yes	No	No	Yes

3	Yes	Yes	No	No	Yes	Borehole	Yes	No	No	Yes

4	Yes	Yes	Yes	No	Yes	Council	Yes	No	No	Yes

**Central Business**										

1	Yes	Yes	Yes	Yes	Yes	Borehole	Yes	Yes	No	Yes

2	Yes	Yes	Yes	No	Yes	Council	Yes	Yes	No	Yes

3	Yes	Yes	Yes	No	Yes	Council	Yes	No	No	Yes

4	No	No	No	No	No	No	No	No	No	No


*Note*: Council water refers to water that is provided by the local authority directly from a centralised water treatment plant. **Source**: Field observations.

**Table 3:** Number of facilities available and required for each market and the state of privacy and cleanliness of the provided facilities.


MARKET CATEGORY AND NUMBER	NUMBER OF STALLS	SANITARY FACILITIES						STATE	

		TOILETS			HAND WASHING				

		AVA	REQ	CM	AVA	REQ	CM	PRIVACY	CLEANLINESS

**Township**									

1	602	9	17	Inad.	1	14	Inad.	No	Not clean

2	442	8	13	Inad.	6	12	Inad.	No	Clean

3	224	2	8	Inad.	1	9	Inad.	No	Clean

4	392	4	11	Inad.	2	10	Inad.	No	Not clean

**Peri-Urban**									

1	490	8	14	Inad.	1	17	Inad.	No	Not clean

2	296	16	9	Adeq.	16	7	Adeq.	No	Clean

3	421	12	13	Inad.	12	13	Inad.	No	Not clean

4	350	4	11	Inad.	2	11	Inad.	No	Not clean

**Central Business**									

1	1970	36	51	Inad.	63	51	Adeq.	No	Clean

2	727	22	20	Inad.	10	20	Inad.	No	Not clean

3	647	10	18	Inad.	2	18	Inad.	No	Clean

4	500	0	14	NP	0	14	NP	N/A	N/A

**Total**	7061	131	196	Adeq.: 1 Inad.: 10 NP: 1	116	196	Adeq.: 2 Inad.: 9 NP: 1	Private: 0 Not Private: 11 NP: 1	Clean: 5 Not clean: 6


**KEY**: NP; Not provided, Ava.; Available, Req.; Required, Adeq.; Adequate, Inad.; Inadequate: Cm.; Comment. **Source**: Field observations.

### Provided sanitary facilities

The observational part of the study revealed that 11 of the 12 selected markets had toilets, hand washing facilities and a water source, i.e., a place to get water for drinking and other use, urinals, and cleaning tissues. Ten markets had washing facilities i.e., showers and or bathtubs, while only four markets had hand drying facilities, sanitary bins and soap (***Table 2***). One of the markets lacked any form of sanitary facilities required by the laws of Zambia.

Many of the markets (10) did not comply with the required number of toilets and hand washing facilities, and six markets did not comply with cleanliness. None of the markets complied with privacy of facilities (***Table 3***). It was observed and reported that some of the traders used unfinished buildings located within the market’s premises as toilets.

### Perception of the traders of provided sanitary facilities

***Table 4*** shows that, respectively, only 27% and 25% of the respondents said that the number of provided toilets and hand washing facilities were adequate. A total of 72% of the traders perceived facilities as not being private. Most of the traders (78%) also said that the facilities were not clean. A high percentage of traders (69%) responded that they only used the facilities due to lack of other options. Most of the traders (61%) perceived the facilities as being located at an appropriate distance from their stalls.

**Table 4:** Perception of traders of the provided sanitary facilities (n = 386).


FACILITY	FREQUENCY(n)	PROPORTION (%)

**Number of Toilets**		

Adequate	104	27

Not adequate	282	73

**Number of Hand Washing Facilities**		

Adequate	96	25

Inadequate	226	58

Not provided	64	17

**Privacy of Facilities**		

Private	108	28

Not private	278	72

**Siting of Facilities**		

Far	148	38

Near	237	61

Missing	1	0.3

**Cleanliness of Facilities**		

Good	84	22

Poor	302	78

**Use of Facilities**		

Always	96	25

Sometimes	267	69

Do not use	23	6


**Source**: Field interviews.

## Discussion

Overall, the findings of the study indicated that although almost all the 12 sampled markets had some form of sanitary facilities for market traders, none of them complied entirely with the laws of Zambia regarding provision of facilities for traders. In addition, most of the market traders perceiving the facilities as not up to standard.

The general inadequacy of sanitary facilities reported in this study and the lack of compliance with Zambian laws on this matter is a potential health hazard to both the traders and to the public. The results of this study are similar with those reported by Sommer et al. [15], who revealed that 74% of marketplaces in Vietnam had no toilets and 13% had inadequate toilets. Our results are also in line with the report by the Nation Reporter [21], which revealed that one of the markets in the Lusaka peri urban area of Zambia was reported to have poor sanitation, in particular noting the absence of toilets. The fact that not all markets had adequate sanitary facilities could be the reason for the observed use of unfinished buildings in the area as toilets. Such poor sanitary conditions may result in diarrhea and other faecal-oral diseases because insects, rodents and other carriers carrying human excreta from these unoccupied buildings can contaminate food at the market. Poor water and sanitation can potentially lead to deaths and disability-adjusted life years lost (DALYs), which have a negative effect on the economy of the country [2,15]. Almedom et al. reported that proper use of toilets can reduce diarrhoea by 36%, thereby illustrating the importance of using toilets [6].

This study showed that most of the traders perceived the sanitary facilities as being unsatisfactory. This might result in traders not using the facilities. Results from a Zambian study on the use of latrines in a community by Thys et al. revealed that people were more comfortable using latrines that were in a good state and had a roof and lockable door for privacy [36]. The participants in the same study also revealed that provision of latrines promoted dignity and respect, and that compared to women, the men were less concerned with provision of latrines and were comfortable with defecating in the open. The poor state of the facilities, as perceived by the traders in the current study, may lead to traders, mostly female, hesitating to use the facilities. Traders may be forced to go outside the market in search of more private and clean facilities, resulting in lost time, as shown in studies conducted in India by Hartmann et al. and Venugopal et al [37,38]. In addition, some of the traders may resort to using bottles and “Shake” packs (opaque beer packs) to answer the call of nature, as reported by the Nation Reporter in Lusaka [21]. The absence of water sources at one of the markets was also hazardous because traders might not always manage to buy safe bottled water for drinking. This may result in dehydration and other heat related disorders, especially during the hot season. In addition, the traders might consider buying cheap drinking water of questionable quality from the street vendors. In the absence of water, the traders might not wash their hands properly after using the toilets, which may in turn lead to diarrhea and other related diseases as reported in a systematic review by Curtis and Cairncross [39].

A study from India Venugopal et al. revealed that the majority (87%) of the women did not have access to toilets at the workplace [38]. Furthermore, the study showed an association between the lack of access to toilets at workplaces and heat-related health symptoms among the women. In this regard, the majority reported periodic genitourinary problems while about 2% of the population had kidney stones, a disease caused by holding back urine. Venugopal et al. further revealed that “Good access to toilet facilities for women at the workplace reduces anxiety and improves the ability to do the work properly, safely, and with concentration, even during their menstrual cycle” [38]. Additionally, 10% of the participants in a study by Venugopal et al. were reported to have stayed at home during their menstrual cycles, which resulted in loss of wages. This present study did not analyse health issues among the traders, but the market traders face the risk of similar health problems due to lack of proper toilet facilities.

Several limitations and strengths were noted in this study. The first limitation is that most of the data in the present study were based on subjective statements obtained by interviews of persons at the market. It is also important to note that the interview guides and checklist for the observation used in the study were not standardized or validated. In addition, selection bias might have been present in this study. In terms of strengths, data was collected through both observations and interviews. The advantage of this combination of methods is that it considers the complexity of human behaviours, thus minimizing research bias. Pretesting was also done to ensure internal validity of the data collection tool. Further, much of the content was extracted from previous studies. Finally, selection bias was reduced by using stratification and random sampling in the selection of markets and traders.

As Zambia and the markets in Zambia are presumably typical of developing countries, this study may provide valuable insights about sanitary facilities in similar countries with similar markets. It is important to improve the state of the facilities in the markets to protect the health of the market traders and the general population. It is essential to ensure that workers have a healthy and safe environment as this has an impact on their productivity, among other things. A major recommendation deriving from this study is that the Lusaka City Council should ensure that all markets are provided with sanitary facilities; more facilities need to be built to reduce inadequacy, and the facilities must be maintained. In addition, the market traders must make sure that they take care of the facilities by seeing to it that they are clean and protected against vandalism.

For the future, analytical in-depth studies that look at the problem of provision of facilities are recommended. Studies of the health status of the workers over time should be performed, along with intervention studies where sanitation in the marketplace is improved. The findings of this study can be generalized to markets in Lusaka district and related settings.

## Conclusions

Overall, the study showed evidence that sanitary facilities in markets of Lusaka district were in very poor condition. Most of the markets did not comply with the laws of Zambia regarding the provision and state of the facilities, such as number of toilets and hand washing facilities, privacy and cleanliness. These observations coincided closely with the traders’ perceptions of the sanitary facilities. These results call for improvement in provision of facilities in markets of Lusaka. There is need to provide adequate facilities and to properly maintain them. The provisions of sanitary facilities will not only improve the health of the traders but the public. This is because markets are meeting places for people from different places. In addition, markets are the main distribution centers for food, entailing contamination of food in the marketplace will put the general population at risk of infection. The results of this study might help public health authorities, including the Lusaka City Council and Ministry of Health, to consider areas needing improvement related to the provision of sanitary facilities in Lusaka markets. In addition, the local authority must improve collection and management of funds paid by customers and traders for use of facilities in the markets. These funds can be used to improve provision and maintenance of facilities in markets. The reward for such efforts will be improved health among market traders, the Lusaka community, and the nation in general.

## Abbreviations

Centre for Disease Control and Prevention (CDC)

Central Statistical Office (CSO)

Millennium Development Goals (MDGs)

Lusaka City Council (LCC)

United Nations International Children’s Emergency Fund (UNICEF)

World Health Organization (WHO)

Zambia National Public Health Institute (ZNPHI)

United Nations Economic Commission for Europe (UNECE)

Government of the Republic of Zambia (GRZ)
